# One-year mortality among Danish intensive care patients with acute kidney injury: a cohort study

**DOI:** 10.1186/cc11420

**Published:** 2012-07-12

**Authors:** Henrik Gammelager, Christian Fynbo Christiansen, Martin Berg Johansen, Else Tønnesen, Bente Jespersen, Henrik Toft Sørensen

**Affiliations:** 1 Department of Clinical Epidemiology, Aarhus University Hospital, Olof Palmes Allé 43-45, Aarhus N, 8200, Denmark; 2 Department of Anesthesiology and Intensive Care Medicine, Aarhus University Hospital, Nørrebrogade 44, Aarhus C, 8000, Denmark; 3 Department of Nephrology, Aarhus University Hospital, Brendstrupgårdsvej 100, Aarhus N, 8200, Denmark

## Abstract

**Introduction:**

There are few studies on long-term mortality among intensive care unit (ICU) patients with acute kidney injury (AKI). We assessed the prevalence of AKI at ICU admission, its impact on mortality during one year of follow-up, and whether the influence of AKI varied in subgroups of ICU patients.

**Methods:**

We identified all adults admitted to any ICU in Northern Denmark (approximately 1.15 million inhabitants) from 2005 through 2010 using population-based medical registries. AKI was defined at ICU admission based on the risk, injury, failure, loss of kidney function, and end-stage kidney disease (RIFLE) classification, using plasma creatinine changes. We included four severity levels: AKI-risk, AKI-injury, AKI-failure, and without AKI. We estimated cumulative mortality by the Kaplan-Meier method and hazard ratios (HRs) using a Cox model adjusted for potential confounders. We computed estimates for all ICU patients and for subgroups with different comorbidity levels, chronic kidney disease status, surgical status, primary hospital diagnosis, and treatment with mechanical ventilation or with inotropes/vasopressors.

**Results:**

We identified 30,762 ICU patients, of which 4,793 (15.6%) had AKI at ICU admission. Thirty-day mortality was 35.5% for the AKI-risk group, 44.2% for the AKI-injury group, and 41.0% for the AKI-failure group, compared with 12.8% for patients without AKI. The corresponding adjusted HRs were 1.96 (95% confidence interval (CI) 1.80-2.13), 2.60 (95% CI 2.38 to 2.85) and 2.41 (95% CI 2.21 to 2.64), compared to patients without AKI. Among patients surviving 30 days (*n *= 25,539), 31- to 365 day mortality was 20.5% for the AKI-risk group, 23.8% for the AKI-injury group, and 23.2% for the AKI-failure group, compared with 10.7% for patients without AKI, corresponding to adjusted HRs of 1.33 (95% CI 1.17 to 1.51), 1.60 (95% CI 1.37 to1.87), and 1.64 (95% CI 1.42 to 1.90), respectively. The association between AKI and 30-day mortality was evident in subgroups of the ICU population, with associations persisting in most subgroups during the 31- to 365-day follow-up period, although to a lesser extent than for the 30-day period.

**Conclusions:**

AKI at ICU admission is an important prognostic factor for mortality throughout the subsequent year.

## Introduction

Acute Kidney Injury (AKI) is defined as an abrupt decline of kidney function, primarily described in recent years using the widely accepted risk, injury, failure, loss of kidney function, and end-stage kidney disease (RIFLE) classification based on changes in serum creatinine level and/or urine output [1,2].

Former studies have reported a prevalence of AKI at ICU admission between 22% and 36% [3-5]. It is associated with 1.4- to 3.2-fold increased in-hospital mortality compared with ICU patients without AKI, depending on the ICU study population and AKI severity [4,5]. ICU studies of the association between maximum AKI level during ICU or hospital stay and hospital mortality have shown similar results [3,6,7]. To date only four ICU studies, with sample sizes from 183 to 10,518 ICU patients with or without AKI, have examined the association between AKI defined by the RIFLE criteria and mortality beyond 90 days [8-11]. These studies have a number of limitations, including patient recruitment at a single center [8-11], inclusion of selected subpopulations of ICU patients (surgical or septic ICU patients) [9-11], lack of adjustment for confounders [8], and loss to follow-up [11].

A large study within a population-based hospital setting with complete history of preadmission comorbidity and complete follow-up is needed to quantify the impact of AKI on long-term mortality, including differential impacts in subgroups of the heterogeneous ICU population. Such information would improve understanding of the clinical course of AKI and identify potentially preventable post-discharge deaths.

We therefore conducted a cohort study to (1) examine the prevalence of AKI at ICU admission, (2) examine its impact on mortality during one year of follow-up, and (3) examine whether the influence of AKI varied in subgroups of ICU patients with different comorbidity levels, chronic kidney disease status, surgical status, primary hospital diagnosis, and treatment with mechanical ventilation, or inotropes/vasopressors.

## Materials and methods

### Setting

We conducted this cohort study using prospectively collected data from medical and administrative registries in Northern Denmark (the former counties of Aarhus and North Jutland, with approximately 1.15 million inhabitants) from 1 January 2005 to 31 December 2011. The Danish National Health Service provides tax-supported health care to all Danish residents, with universal access to public hospitals and general practitioners. All intensive care in Denmark is provided at these public hospitals. The unique 10-digit civil registration number assigned to all Danish residents since 1968 permits unambiguous linkage between medical databases [12].

Nearly all medical treatments, except very few highly specialized treatments (for example, liver transplantation and lung transplantation) are provided in the study region, which has twelve ICUs, eight at university hospitals and four at regional hospitals.

### ICU patients

We identified all adult residents (aged 15 years or older) with a first-time ICU admission from 1 January 2005 to 31 December 2010 using the Danish National Registry of Patients (DNRP) [13]. We required one-year residency in the study region before the index hospitalization to ensure availability of data on previous laboratory measurements from the laboratory database. It is mandatory for hospitals in Denmark to electronically report information on all hospital contacts to the DNRP. The DNRP includes data from non-psychiatric hospital admissions since 1977. Since 1995, the registry has also covered all emergency room and outpatient clinic visits. Data in the registry include civil registration numbers, emergency vs. planned hospital admission, dates of hospital admission and discharge, hospital and department, surgical procedures and major treatments performed, one primary discharge diagnosis (main reason for hospitalization) and up to 19 secondary discharge diagnoses. Since 1994, diagnoses have been coded using the *International Classification of Diseases*, 10^th ^revision (ICD-10) [14]. Information on ICU admissions and major treatments during the ICU stay, such as mechanical ventilation, acute renal replacement therapy, and treatment with inotropes/vasopressors, have been coded in the DNRP with a high degree of accuracy since 2005 [13].

We used the primary ICD-10 diagnosis for the current hospitalization to classify patients into nine disease categories as a proxy for reason for ICU admission, which is not recorded in the DNRP. In addition, we specified five types of ICU admission: non-surgical, elective non-cardiac surgical, elective cardiac surgical, acute non-cardiac surgical and acute cardiac surgical. We used the Nordic Medico-Statistical Committee classification of surgical procedures in the DNRP to classify patients as cardiac and non-cardiac surgical based on whether they had any surgical procedure and on type of surgical procedure up to 7 days before or on the day of ICU admission, respectively [15]. Surgical ICU patients were further divided into acute or elective, according to the hospital admission type registered in the DNRP. Admission type is recorded with high accuracy in the DNRP [14].

### Acute kidney injury

The laboratory database covering the study area contains laboratory tests from all inpatient stays, outpatient clinic visits, and visits to general practitioners [16]. We searched the laboratory database for the highest measurement of plasma creatinine, which is equivalent to serum creatinine [17], on the day of ICU admission. For patients with missing values on that day, we calculated the mean of the highest creatinine measurements available on the day before and the day after ICU admission [18]. We used the creatinine level to classify each patient into one of three AKI severity levels based on the RIFLE criteria: AKI-risk defined as a 50% to 100% increase in creatinine from the baseline level, AKI-injury defined as a 100% to 200% increase, and AKI-failure defined as an increase of 200% or more or creatinine values ≥ 354 μmol/l, with an acute rise > 44 μmol/l up to seven days before ICU admission [1]. All other ICU patients were classified as without AKI. Baseline creatinine was defined as the most recent creatinine measurement from an outpatient clinic or general practitioner in the period from one year to seven days before the current hospitalization [19]. Creatinine assessments up to seven days before the current hospitalization were not considered, because the AKI process may have started before hospital admission. For patients lacking a measured baseline creatinine level and without chronic kidney disease (CKD), we estimated baseline creatinine using the four-variable version of the Modification of Diet in Renal Disease (MDRD) equation based on age, race, and gender assuming a normal glomerular filtration rate (GFR) of 75 ml/min, as suggested in the RIFLE criteria [1]. We assumed that all patients were Caucasians. Patients receiving chronic dialysis treatment, those with a previous kidney transplant, and those lacking information on creatinine level on the day of ICU admission, and on the day before and the day after admission were excluded from the study.

### Covariates

We obtained data on preexisting comorbidity based on inpatient and outpatient diagnoses five years before the current hospitalization and used these to compute the Charlson Comorbidity Index (CCI) scores [20]. Patients were categorized as having low (score = 0), medium (score 1 to 2) and high (score ≥ 3) levels of comorbidity [21,22]. Kidney diseases were excluded from the CCI and addressed separately because the exposure under study was kidney dysfunction. CKD was included as a covariate, defined as an estimated GFR (eGFR) below 60 ml/min per 1.73 m^2 ^using the four-variable MDRD equation (stage 3 or higher CKD according to National Kidney Foundation guidelines) [23]. We used the most recent plasma creatinine measurement from an outpatient clinic or general practitioner one year to seven days before the current hospitalization to compute eGFR [19]. In addition, we also computed the length of the entire hospital stay, including continuous hospitalizations with inter-hospital transfer. All relevant codes are provided in Additional file 1.

### Follow-up for mortality

Deaths and migration were identified from the Danish Civil Registration System through 31 December 2011. This registration system is updated daily and contains complete information since 1968 on migration, vital status, and the exact date of death (when relevant) for all Danish citizens [24].

### Statistical analysis

Patient characteristics, including demographic characteristics, preexisting comorbidity level, and information from the current hospitalization, were tabulated by RIFLE group.

We followed patients from ICU admission until death or emigration, or for up to one year, whichever came first. The Kaplan-Meier method was used to compute mortality function curves (1 - survival function) and to estimate cumulative mortality for three time periods: 0 to 30 days, 31 to 365 days, and 0 to 365 days following ICU admission. We computed hazard ratios (HRs) within 0- to 30-day and 31- to 365-day periods using Cox proportional hazards regression, controlling for age, gender, CKD, CCI level, and surgical status. The assumption of proportional hazards was checked graphically using log(-log(survival probability)) plots and was found appropriate.

To examine potentially differing effects of AKI on mortality in subgroups of ICU patients (effect measure modification) [25], we stratified the analyses by age groups, CCI levels, surgical status, CKD, primary hospital diagnosis, and treatment with mechanical ventilation or inotropes/vasopressors. In these subgroup analyses we combined patients with any degree of AKI into one group.

We conducted a sensitivity analysis to examine the potential influence of excluding patients lacking a creatinine measurement at ICU admission. In this analysis we estimated AKI levels for patients with missing creatinine using multiple imputations [26-28], generating five imputed datasets. HRs were calculated as the average HRs of the five datasets, corrected for between- and within-imputation variation [26-28]. The imputation model included all measured covariates in Table 1, the outcome, and the Nelson-Aalen estimator of the cumulative baseline hazard evaluated at the observed survival time [29].

**Table 1:** Characteristics by AKI level among 30,762 ICU patients, Northern Denmark, 2005 to 2010

	Without AKI *n *= 25,969 (84.4%)	AKI-risk *n *= 1,986 (6.5%)	AKI-injury *n *= 1,311 (4.3%)	AKI-failure *n *= 1,496 (4.9%)
**Age**				
Median age (IQR)	64 (49, 75)	72 (61, 80)	71 (59, 80)	69 (59, 78)
**Gender**				
Female	11,172 (43.0%)	878 (44.2%)	645 (49.2%)	657 (43.9%)
Male	14,797 (57.0%)	1,108 (55.8%)	666 (50.8%)	839 (56.1%)
**Charlson comorbidity index score^a^**				
Low (score 0)	13,862 (53.4%)	798 (40.2%)	510 (38.9%)	556 (37.2%)
Medium (score 1 to 2)	8,654 (33.3%)	804 (40.5%)	507 (38.7%)	579 (38.7%)
High (score ≥ 3)	3,453 (13.3%)	384 (19.3%)	294 (22.4%)	361 (24.1%)
**Chronic kidney disease^b^**				
Yes	3,283 (12.6%)	395 (19.9%)	199 (15.2%)	470 (31.4%)
No	22,686 (87.4%)	1,591 (80.1%)	1,112 (84.8%)	1,026 (68.6%)
**Surgical status^c, d^**				
Non-surgical	9,495 (36.6%)	892 (44.9%)	593 (45.2%)	786 (52.5%)
Surgical				
Acute non-cardiac	8,271 (31.8%)	752 (37.9%)	564 (43.0%)	554 (37.0%)
Acute cardiac	920 (3.5%)	102(5.1%)	40 (3.1%)	34 (2.3%)
Elective non-cardiac	3,939 (15.2%)	188 (9.5%)	101 (7.7%)	106 (7.1%)
Elective cardiac	3,344 (12.9%)	52 (2.6%)	13 (1.0%)	16 (1.1%)
**Primary diagnosis during current hospitalization**				
Septicemia	232 (0.9%)	100 (5.0%)	127 (9.7%)	187 (12.5%)
Other infectious diseases	2,329 (9.0%)	254 (12.8%)	182 (13.9%)	194 (13.0%)
Endocrinology diseases	359 (1.4%)	65 (3.3%)	52 (4.0%)	82 (5.5%)
Cardiovascular diseases	7.377 (28.4%)	464 (23.4%)	196 (15.0%)	183 (12.2%)
Respiratory diseases	1,427 (5.5%)	180 (9.1%)	77 (5.9%)	66 (4.4%)
Gastrointestinal or liver diseases	2,416 (9.3%)	322 (16.2%)	266 (20.3%)	239 (16.0%)
Cancer or other neoplasm	3.410 (13.1%)	175 (8.8%)	139 (10.6%)	130 (8.7%)
Trauma or poisoning	4,651 (17.9%)	168 (8.5%)	110 (8.4%)	96 (6.4%)
Other	3,758 (14.5%)	258 (13.0%)	162 (12.4%)	319 (21.3%)
**Laboratory information**				
Baseline creatinine measured	17,384 (66.9%)	1,530 (77.0%)	950 (72.5%)	1.164 (77.8%)
ICU admission creatinine, μmol/L (IQR)	75 (61, 92)	137 (112, 168)	190 (153, 230)	375 (280, 516)
**ICU treatments**				
Acute renal replacement therapy	482 (1.9%)	206 (10.4%)	220 (16.8%)	561 (37.5%)
Mechanical ventilation	9,673 (37.2%)	965 (48.6%)	697 (53.2%)	719 (48.1%)
Inotropes/vasopressors	7,823 (30.1%)	939 (47.3%)	756 (57.7%)	864 (57.8%)
**Length of admission**				
In-hospital days, median (IQR)	10 (4, 19)	13 (5, 26)	14 (5, 30)	16 (6, 33)
In-hospital days before ICU admission, median (IQR)	1 (0, 2)	1 (0, 3)	1 (0, 3)	1 (0, 3)

Results are presented as number (percent) of patients unless stated otherwise.

^a^Charlson comorbidity index score after exclusion of kidney diseases.

^b^eGFR < 60 ml/min per 1.73 m^2^.

^c^Surgical status and cardiac surgical status identified by surgery and type of surgery on or up to 7 days before ICU admission.

^d^Acute and elective status classified according to hospital admission type.

AKI, acute kidney injury; CI, confidence interval; ICU, intensive care unit; IQR, interquartile range.

Analyses were performed using the statistical software package Stata version 11.0 (StataCorp LP, College Station; TX, USA). All data were obtained from Danish registries, which are generally available to researchers, and their use does not require ethical approval or informed consent. The study was approved by the Danish Data Protection Agency (record number 2009-41-3987).

## Results

### Descriptive data

The study population comprised 30,762 adults admitted to an ICU in Northern Denmark during the six-year observation period, after excluding 192 (0.6%) patients receiving chronic dialysis or with a previous kidney transplant, and 1,578 (4.9%) patients lacking information on plasma creatinine level at ICU admission. Patients without a creatinine measurement were younger and had less preexisting comorbidity and shorter hospital stays compared with patients with a creatinine measurement (Additional file 2). The total time of follow up was 23,850 person years (median duration 365 days, interquartile range 258 to 365).

The median age in the study population was 65 years and 13,352 (43%) patients were female. At ICU admission, 4,793 (15.6%) patients had AKI; these included 1,986 (6.5%) patients with AKI-risk, 1,311 (4.3%) with AKI-injury, and 1,496 (4.9%) with AKI-failure. Preadmission baseline plasma creatinine results were available for 21,028 (68.4%) patients, and were estimated using the MDRD equation for the remaining 9,734 (31.6%) patients.

Patients with AKI were older and had more preexisting comorbidity, including CKD, than other ICU patients (Table 1). The most frequent diagnoses among AKI patients were other infectious disease, gastrointestinal or liver disease, and cardiovascular disease. AKI was less frequent in elective surgical patients (cardiac and non-cardiac) compared with both non-surgical and acute surgical patients (cardiac and non-cardiac). In addition, patients with AKI were more often treated with mechanical ventilation, inotropes/vasopressors, and as expected, with dialysis during their ICU stay compared to patients without AKI (patients without AKI 1.9%, patients with AKI-risk 10.4%, patients with AKI-injury 16.8%, and patients with AKI-failure 37.5%) (Table 1).

During the time between ICU admission and hospital discharge (median duration 8 days, interquartile range 3 to 17), another 3,099 (10.1%) patients developed AKI.

### Mortality

**Figure 1:**
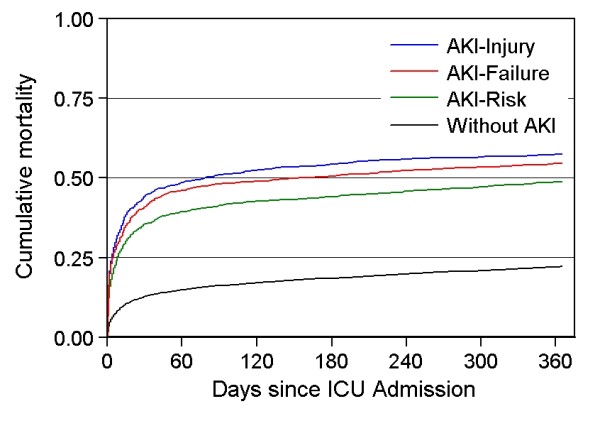
**Cumulative one-year mortality by AKI level, Northern Denmark, 2005 to 2010**.

The one-year mortality was 48.7% (95% confidence interval (CI) 46.5% to 50.9%) for the AKI-risk group, 57.4% (95% CI 54.8% to 60.1%) for the AKI-injury group and 54.7% (95% CI 52.1% to 57.2%) for the AKI-failure group, compared with 22.1% (95% CI 21.6% to 22.7%) for the patients without AKI (Figure 1).

#### Overall 0- to 30-day mortality

Thirty-day mortality was 35.5% (95% CI 33.4% to 37.6%) for the AKI-risk group, 44.2% (95% CI 41.5% to 46.9%) for the AKI-injury group, and 41.0% (95% CI 38.5% to 43.5%) for the AKI-failure group, compared with 12.8% (95% CI 12.4% to 13.2%) for patients without AKI. This corresponded to adjusted HRs of 1.96 (95% CI 1.80 to 2.13), 2.60 (95% CI 2.38 to 2.85), and 2.41 (95% CI 2.21 to 2.64), respectively, all compared with ICU patients without AKI (Table 2).

**Table 2:** Cumulative 30-day and 31-365 day mortality and corresponding hazard ratios (HRs) by AKI status

	Number of deaths	Number at period start	Cumulative mortality % (95% CI)	Unadjusted HR (95% CI)	Adjusted HR^a^ (95% CI)
**0 to 30 days**					
Without AKI	3,327	25,969	12.8% (12.4-13.2)	1(ref.)	1(ref.)
AKI-risk	704	1,986	35.5% (33.4-37.6)	3.17 (2.93-3.45)	1.96 (1.80-2.13)
AKI-injury	579	1,311	44.2% (41.5-46.9)	4.21 (3.86-4.60)	2.60 (2.38-2.85)
AKI-failure	613	1,496	41.0% (38.5-43.5)	3.83 (3.52-4.18)	2.41 (2.21-2.64)
**31 to 365 days**					
Without AKI	2,421	22,642	10.7% (10.3-11.1)	1 (reference)	1 (reference)
AKI-risk	263	1,282	20.5% (18.4-22.8)	2.04 (1.80-2.32)	1.33 (1.17-1.51)
AKI-injury	174	732	23.8% (20.9-27.0)	2.46 (2.11-2.87)	1.60 (1.37-1.87)
AKI-failure	205	883	23.2% (20.6-26.1)	2.38 (2.06-2.75)	1.64 (1.42-1.90)

^a^Adjusted for age, gender, Charlson comorbidity index score, surgical status, and chronic kidney disease.

AKI, acute kidney injury; CI, confidence interval; HR, hazard ratio; ICU, intensive care unit.

#### Overall 31- to 365-day mortalitys

Among patients surviving 30 days (*n *= 25,539), mortality between 31 days and 365 days was 20.5% (95% CI 18.4% to 22.8%) for the AKI-risk group, 23.8% (95% CI 20.9% to 27.0%) for the AKI-injury group, and 23.2% (95% CI 20.6% to 26.1%) for the AKI-failure group compared with 10.7% (95% CI 10.3% to 11.1%) for patients without AKI. The adjusted HRs were 1.33 (95% CI 1.17 to 1.51), 1.60 (95% CI 1.37 to 1.87), and 1.64 (95% CI 1.42 to 1.90), respectively, compared with ICU patients without AKI (Table 2).

#### Subgroup analyses

The association between AKI and 30-day mortality was evident in all subgroups of the ICU population (Table 3). The relative impact of AKI was most pronounced in patients aged 15 to 40 years; the mortality of patients without AKI in this subgroup was 2.5%, compared to 16.8% for patients with any degree of AKI. This corresponds to an adjusted HR of 4.87 (95% CI 3.33 to 7.13) (Table 3). The relative impact of AKI was also more pronounced among both elective cardiac and non-cardiac surgical patients and among acute cardiac surgical patients, with adjusted HRs (95% CIs) of 3.76 (1.62 to 8.77), 3.43 (2.65 to 4.45), and 3.27 (2.48 to 4.31), respectively, and among patients with low CCI scores (adjusted HR 2.55, 95% CI 2.31 to 2.81), due to a low baseline hazard. By diagnostic category, the adjusted HRs ranged from 1.53 (95% CI 1.19 to 1.96) among patients with a primary registry diagnosis of septicemia to 2.54 (95% CI 2.20 to 2.93) for patients with a primary diagnosis of gastrointestinal or liver disease and 2.59 (95% CI 2.12 to 3.16) for cancer patients. The association between AKI and 30-day mortality was also evident in patients treated with mechanical ventilation (adjusted HR 1.60, 95% CI 1.48 to 1.72) or inotropes/vasopressors (adjusted HR 1.77, 95% CI 1.63 to 1.91) and in patients with CKD (adjusted HR 1.80, 95% CI 1.60 to 2.02).

**Table 3:** Cumulative 30-day mortality and corresponding adjusted hazard ratios (HRs)

		Without AKI		With AKI	
		
	Number	Cumulative mortality % (95% CI)	Adjusted HR (95% CI)	Cumulative mortality % (95% CI)	Adjusted HR^a^ (95% CI)
**Overall**	30,762	12.8 (12.4-13.2)	1 (reference)	39.6 (38.2-41.0)	2.27 (2.14-2.40)
**Age group, years**					
≥ 15 < 40	4,670	2.5 (2.1-3.0)	1 (reference)	16.8 (12.8-21.9)	4.87 (3.33-7.13)
≥ 40 < 60	7,397	7.6 (7.0-8.3)	1 (reference)	29.2 (26.4-32.2)	3.18 (2.72-3.70)
≥ 60 < 80	14,184	14.1 (13.5-14.8)	1 (reference)	39.1 (37.2-41.1)	2.28 (2.11-2.47)
≥ 80	4,511	31.4 (29.9-33.0)	1 (reference)	55.1 (52.2-58.1)	1.83 (1.65-2.02)
**Charlson comorbidity index score^a^**					
Low (score 0)	15,726	9.0 (8.5-9.4)	1 (reference)	35.0 (32.9-37.2)	2.55 (2.31-2.81)
Medium (score 1 to 2)	10,544	15.7 (15.0-16.5)	1 (reference)	41.4 (39.2-43.6)	2.17 (1.99-2.38)
High (score ≥ 3)	4,492	21.1 (19.8-22.5)	1 (reference)	44.5 (42.0-47.5)	1.96 (1.74-2.20)
**Surgical status^c, d^**					
Non-surgical	11,766	16.7 (16.0-17.5)	1 (reference)	41.7 (39.7-43.7)	2.01 (1.85-2.18)
Surgical					
Acute non-cardiac	10,141	15.9 (15.2-16.7)	1 (reference)	42.0 (40.8-44.3)	2.40 (2.20-2.63)
Acute cardiac	1,096	15.8 (13.6-18.3)	1 (reference)	44.3 (37.3-52.0)	3.27 (2.48-4.31)
Elective non-cardiac	4,334	5.4 (4.8-6.2)	1 (reference)	20.5 (16.9-24.8)	3.43 (2.65-4.45)
Elective cardiac	3,425	1.9 (1.5-2.4)	1 (reference)	7.4 (3.4-5.8)	3.76 (1.62-8.77)
**Primary diagnosis during current hospitalization**					
Septicemia	646	38.8 (32.9-45.4)	1 (reference)	52.2 (47.5-57.1)	1.53 (1.19-1.96)
Other infectious diseases	2,959	15.0 (13.6-16.5)	1 (reference)	36.5 (32.9-40.4)	1.97 (1.66-2.33)
Endocrinology diseases	558	7.2 (5.0-10.5)	1 (reference)	17.1 (12.5-23.1)	1.89 (1.11-3.19)
Cardiovascular diseases	8,220	13.8 (13.0-14.6)	1 (reference)	44.3 (41.0-47.7)	2.14 (1.89-2.41)
Respiratory diseases	1,750	27.8 (25.5-30.2)	1 (reference)	47.1 (41.8-52.7)	1.76 (1.46-2.13)
Gastrointestinal or liver diseases	3,243	17.1 (15.6-18.6)	1 (reference)	41.7 (38.4-45.2)	2.54 (2.20-2.93)
Cancer or other neoplasm	3,854	10.3 (9.3-11.4)	1 (reference)	34.5 (30.2-39.1)	2.59 (2.12-3.16)
Trauma or poisoning	5,035	7.6 (6.9-8.4)	1 (reference)	32.6 (28.1-37.6)	2.41 (1.95-2.99)
Other	4,497	8.8 (8.0-9.8)	1 (reference)	36.7 (33.3-40.3)	2.62 (2.22-3.09)
**Chronic kidney disease^b^**					
Yes	4,347	24. 1 (22.7-25.6)	1 (reference)	44.3 (41.3-47.3)	1.80 (1.60-2.02)
No	26,415	11.2 (10.7-11.6)	1 (reference)	38.2 (36.7-39.8)	2.45 (2.30-2.63)
**ICU treatments**					
Mechanical ventilation	12,054	20.5 (19.5-21.6)	1 (reference)	46.2 (44.2-48.2)	1.60 (1.48-1.72)
Inotropes/vasopressors	10,382	19.3 (18.4-20.2)	1 (reference)	46.2 (44.3-48.2)	1.77 (1.63-1.91)

^a^Compared to patients without AKI within subgroups and adjusted for age, gender, Charlson comorbidity index score, surgical status, and chronic kidney disease.

^b^eGFR < 60 ml/min per 1.73 m^2^.

^c^Surgical status and cardiac surgical status identified by surgery and type of surgery on or up to 7 days before ICU admission, respectively.

^d^Acute and elective status classified according to hospital admission type

AKI, acute kidney injury; CI, confidence interval; HR, hazard ratio; ICU, intensive care unit.

After 30 days of follow-up, AKI still was associated with increased mortality in most subgroups, although to a less pronounced degree than in the 30-day period after ICU admission (Table 4).

**Table 4:** Cumulative 31- to 365-day mortality and corresponding adjusted hazard ratios (HRs)

	Number	Without AKI		With AKI	
		
		Cumulative mortality % (95%CI)	Adjusted HR (95%CI)	Cumulative mortality % (95%CI)	Adjusted HR^a^ (95%CI)
**Overall**	25,539	10.7 (10.3-11.1)	1 (reference)	22.2 (20.7-23.7)	1.49 (1.36-1.63)
**Age group, years**					
≥ 15 < 40	4,516	1.7 (1.4-2.1)	1 (reference)	5.1 (2.8-8.9)	1.52 (0.79-2.94)
≥ 40 < 60	6,627	7.5 (6.9-8.2)	1 (reference)	15.4 (12.9-18.3)	1.58 (1.27-1.97)
≥ 60 < 80	11,561	13.8 (13.2-14.5)	1 (reference)	23.8 (21.7-26.0)	1.41 (1.25-1.59)
≥ 80	2,835	21.8 (20.2-23.6)	1 (reference)	34.2 (30.1-38.5)	1.46 (1.22-1.74)
**Charlson comorbidity index score**					
Low (score 0)	13,834	5.5 (5.1-5.9)	1 (reference)	15.3 (13.4-17.4)	1.88 (1.59-2.22)
Medium (score 1 to 2)	8,403	14.5 (13.7-15.4)	1 (reference)	25.8 (23.3-28.5)	1.60 (1.40-1.83)
High (score ≥ 3)	3,302	24.6 (23.0-26.2)	1 (reference)	29.6 (26.1-33.5)	1.12 (0.94-1.34)
**Surgical status^c, d^**					
Non-surgical	9,234	10.3 (9.6-10.9)	1 (reference)	22.1 (20.0-24.5)	1.42 (1.24-1.63)
Surgical					
Acute non-cardiac	8,038	13.1 (12.4-13.9)	1 (reference)	23.97 (21.5-26.5)	1.46 (1.27-1.68)
Acute cardiac	873	5.6 (4.2-7.4)	1 (reference)	21.4 (14.5-30.9)	4.44 (2.63-7.51)
Elective non-cardiac	4,039	14.3 (13.2-15.4)	1 (reference)	19.1 (15.2-23.9)	1.28 (0.98-1.67)
Elective cardiac	3.355	3.8 (3.2-4.5)	1 (reference)	12.0 (6.4-21.8)	2.96 (1.50-5.85)
**Primary diagnosis during current hospitalization**					
Septicemia	340	21.8 (15.9-29.6)	1 (reference)	17.7 (13.0-23.7)	0.81 (0.49-1.32)
Infectious diseases	2,380	10.0 (8.8-11.4)	1 (reference)	19.8 (16.2-24.0)	1.28 (1.02-1.61)
Endocrinology diseases	498	10.2 (7.4-14.0)	1 (reference)	12.1 (8.0-18.2)	0.85 (0.48-1.50)
Cardiovascular diseases	6,829	6.2 (5.7-6.9)	1 (reference)	20.9 (17.5-24.8)	2.21 (1.76-2.78)
Respiratory diseases	1,202	22.0 (19.6-24.7)	1 (reference)	31.6 (25.2-39.1)	1.27 (0.94-1.73)
Gastrointestinal or liver diseases	2,486	14.0 (12.6-15.6)	1 (reference)	25.3 (21.7-29.4)	1.75 (1.41-2.17)
Cancer or other neoplasm	3,350	24.5 (23.0-26.0)	1 (reference)	32.0 (26.9-37.7)	1.17 (0.93-1.46)
Trauma or poisoning	4,560	5.3 (4.6-6.0)	1 (reference)	20.7 (16.2-26.2)	1.85 (1.36-2.53)
Other	3,894	8.1 (7.3-9.1)	1 (reference)	19.0 (15.7-22.9)	1.37 (1.08-1.75)
**Chronic kidney disease^b^**					
Yes	3,084	19.5 (18.0-21.1)	1 (reference)	28.7 (25.2-32.5)	1.43 (1.19-1.71)
No	22,455	9.6 (9.2-10.0)	1 (reference)	20.5 (18.9-22.2)	1.48 (1.33-1.64)
**ICU treatments**					
Mechanical ventilation	8,976	10.6 (10.0-11.4)	1 (reference)	24.6 (22.3-27.0)	1.49 (1.30-1.70)
Inotropes/vasopressors	7,691	12.3 (11.5-13.2)	1 (reference)	24.7 (22.5-27.1)	1.46 (1.27-1.66)

^a^Compared to patients without AKI within subgroups and adjusted for age, gender, Charlson comorbidity index score, surgical status, and chronic kidney disease.

^b^eGFR < 60 ml/min per 1.73 m^2^.

^c^Surgical status and cardiac surgical status identified by surgery and surgical type on or up to 7 days before ICU admission, respectively.

^d^Acute and elective status classified according to hospital admission type.

AKI, acute kidney injury; CI, confidence interval; HR, hazard ratio; ICU, intensive care unit.

### Sensitivity analysis

The associations between AKI and mortality were similar after imputation of missing creatinine measurement at ICU admission. The adjusted 30-day HRs were 1.95 (95% CI 1.80 to 2.12) for the AKI-risk group, 2.62 (95% CI 2.39 to 2.86) for the AKI-injury group and 2.42 (95% CI 2.22 to 2.64) for the AKI-failure group. In the period 31 to 365 days following ICU admission, the adjusted HRs were 1.36 (95% CI 1.19 to 1.54), 1.61 (95% CI 1.38 to 1.88), 1.66 (95% CI 1.43 to 1.92) for the AKI-risk, AKI-injury, and AKI-failure groups, respectively.

## Discussion

In this large cohort study conducted within a population-based hospital setting, we found that 15% of ICU patients had AKI at ICU admission. AKI at ICU admission was associated with a two-fold increased 30-day mortality for patients in the AKI-risk group and two-and-a-half-fold increased 30-day mortality in the AKI-injury and AKI-failure groups. Relative mortality in AKI patients remained elevated, with 33% to 64% increased mortality during the 31- to 365-day period following ICU admission. The relative impact of AKI on 30-day mortality was most pronounced in younger age groups and among elective surgical and acute cardiac surgical ICU patients.

### Existing studies

Our study extends current knowledge by providing complete one-year mortality information and by examining the differential impact of AKI on mortality in subgroups of the ICU population in a population-based setting.

Previous studies reported a higher prevalence of RIFLE-defined AKI at the time of ICU admission (22% to 36%) compared to our findings [3-5]. This may stem from heterogeneity in study cohorts and from estimation of baseline creatinine by assuming GFR of 75 ml/min in cohorts including patients with CKD [4,5], which may overestimate the prevalence of AKI [30].

In accordance with our findings of increased short-term mortality, five recent large studies with between 5,000 and 120,000 ICU patients all reported increased in-hospital mortality among patients with RIFLE-defined AKI at ICU admission or during an ICU stay, compared with ICU patients without AKI [3-7]. In these studies, relative risk of in-hospital mortality ranged from 1.0 to 1.6 among patients with AKI-risk, from 1.4 to 4.0 among patients with AKI-injury, and from 1.6 to 4.1 among patients with AKI-failure.

None of these studies included follow-up after hospital discharge. Similar to our results, two studies found that the impact of AKI on short-term mortality was similar in the AKI-injury and AKI-failure groups [6,7]. The variation found in the relative impact of AKI on short-term mortality in our study vs. and among previous studies may be explained by the heterogeneity of study cohorts, use of estimated vs. measured baseline creatinine levels, examination of the most advanced AKI stage during an ICU stay vs. AKI level at ICU admission, availability of data on urine output for the RIFLE classification, different approaches to adjusting for potential confounders, and examination of in-hospital mortality compared to mortality at 30 days or another fixed time point [30-32]. None of the five large earlier studies reported results from subgroups of the ICU population [3-7].

To our knowledge, only four studies have examined the association of AKI defined by the RIFLE criteria and long-term mortality (beyond 90 days) in ICU patients. All were single center studies [8-11]. These studies also observed increased long-term mortality among ICU patients with AKI compared with patients without AKI. Our finding of increased 30- to 365-day mortality among both acute and elective surgical ICU patients is in line with the work of Bihorac *et al. *who examined a cohort of 10,518 elective and acute surgical ICU patients discharged from an American hospital [9]. They reported the following 10-year adjusted HRs after hospitalization: 1.18 (95% CI 1.08 to 1.29) for patients with AKI-risk, 1.43 (95% CI 1.29 to 1.59) for patients with AKI-injury, and 1.57 (95% CI 1.40 to 1.75) for patients with AKI-failure, compared to patients without AKI [9]. They reported similar relative estimates in a study restricted to elective and acute cardiothoracic surgical ICU patients [10]. The slight difference between the results from these two studies and our results may primarily be a result of a different composition of elective and acute surgery as well as type of surgical procedures in the cohorts of ICU patients. A Spanish study of 234 ICU patients with sepsis who survived to hospital discharge, found a relative risk of two-year mortality of 3.2 (95% CI 1.6 to 6.5) in patients with AKI during the ICU stay compared to patients without AKI. However, data on mortality were not available for 23% of the initial cohort [11], and may not be directly comparable with our subgroup of patients with a primary hospital diagnosis of septicemia in which we found no impact of AKI on 31- to 365-day mortality. In a UK cohort study of 153 patients with AKI at ICU admission, Abosaif *et al. *observed 6-month mortality of 43.3%, 53.6% and 86.0% for patients with AKI-risk, AKI-injury, and AKI-failure, respectively [8]. The crude 6-month mortality risk for patients with AKI-risk and AKI-injury corresponds well with our findings.

### Strengths and limitations

The main strengths of our study include its large size, well-defined study population, uniform access to health care in Denmark, comprehensive laboratory data including baseline measurements from outpatient clinics and general practitioners, and complete follow-up data. However, several additional issues should be considered when interpreting our results.

First, we used routine laboratory data to assess AKI at ICU admission, and some creatinine measurements (that is, those measured only with an arterial blood gas analyzer in the ICU) may not be transferred to the laboratory database. We excluded patients without a creatinine measurement at ICU admission. However, the overall results did not change after imputation of AKI level. Second, as our routine data did not include information about urine output we could not utilize urine criteria in the RIFLE classification of AKI. However, urine output criteria are affected by diuretics, which are commonly used in ICU patients. Third, we assessed AKI severity at ICU admission, when follow-up commenced. We thereby avoided including follow-up time before fulfilment of AKI criteria, that is, immortal person-time [33,34]. However this limits the generalization to patients with AKI at ICU admission. Fourth, we did not have detailed data on severity of illness scores at ICU admission or during the ICU stay. The physiological variables included in these scores may be part of the causal pathway and adjustment may thereby attenuate any true association [35]. Still, the impact of AKI on mortality was evident in subgroups of ICU patients treated with mechanical ventilation and inotropes/vasopressors, which may be indicators of more severe illness. In general, correct selection of confounders in prognostic studies of cohorts of ICU patients is challenging. Many covariates may be part of the causal pathway from exposure to outcome. Finally, despite adjustment for potential confounders, we cannot rule out unmeasured and residual confounding.

## Conclusions

In this large cohort study, AKI was present at ICU admission in 15% of adult ICU patients. Any degree of AKI at ICU admission was associated with markedly increased 30-day mortality and the association was still evident in the 31- to 365-day period. The association was also robust in subgroups of ICU patients, with only slight variation.

## Key messages

• The increased risk of death in patients with AKI at ICU admission was evident throughout the first year after ICU admission.

• The association was evident regardless of age, CKD, preexisting comorbidity, diagnostic category, and surgical status.

• The relative 30-day mortality was highest in younger age groups, elective surgical ICU patients, and acute cardiac surgical ICU patients.

## Abbreviations

AKI: acute kidney injury; CCI: Charlson comorbidity index; CI: confidence interval; CKD: chronic kidney disease; DNRP: Danish National Registry of Patients; eGFR: estimated glomerular filtration rate; HR: hazard ratio; ICD: International Classification of Diseases; ICU: intensive care unit; IQR: inter quartile range; RIFLE: risk, injury, failure, loss of kidney function, and end-stage kidney disease.

## Competing interests

The authors declare that they have no competing interests.

## Authors' contributions

HTS, CFC, and HG conceived the study idea. HG, CFC, BJ, MBJ and HTS designed the study. MBJ and HTS collected the data. HG and MBJ analyzed the data. All authors interpreted the findings. HG and CFC reviewed the literature. HG wrote the first draft, and all authors critically reviewed and edited the manuscript and approved the final version.

## Supplementary Material

Additional file 1 **List of relevant codes used in the current study**. Click here for file

Additional file 2 **Table describing the characteristics of patients with and without a creatinine measurement on the day of ICU admission, and on the day before and the day after admission**. Click here for file
