# Supplementary material for: One-year mortality among Danish intensive care patients with acute kidney injury: a cohort study
Source: Crit Care. 2012 Jul 12;16(4):R124. doi: 10.1186/cc11420 (PMC3580703; doi:10.1186/cc11420)
Supplement: Additional file 1 — List of relevant codes used in the current study. [file cc11420-S1.PDF]

Relevant codes used in the current study

Danish treatment codes defining ICU admission:

- Intensive observation: NABE
- Intensive therapy: NABB

Nomenclature, Properties and Units in Laboratory Medicine (NPU) codes and local Danish laboratory codes used to identify creatinine measurements in the laboratory database:

- NPU18016; NPU01807; NPU04998; ASS00356; ASS00354; ASS00355

International Classification of Diseases (ICD)-10 codes defining primary diagnosis during current hospitalization:

- Septicemia: A02.1, A32.7, A39.2, A40-A41, A.42.7, B37.7
- Other infectious diseases :A00-B99 (except: A02.1, A32.7, A39.2, A40-A41, A.42.7, B37.7),, G00-G07, I00-I02, I30.1, I32.0, I33, I38, I40.0, J00-J06, J36, J39.0, J10-J22, J85.1, J86, K35, K37, K57.0, K57.2, K57.4, K57.8, K61, K63.0, K65.0, K65.9, K67, K75.0, K75.1, K80.0, K80.3, K80.4, K81.0, K81.9, K83.0, L00-L03, L05-L08, M00, M01, M86, N10, N12, N15.1, N30, N39.0, N41, N45, N70-N77
- Cancer or other neoplasm: C00-D89
- Endocrine diseases E00-E90
- Cardiovascular diseases: I00-I99 without I00-I02, I30.1, I32.0, I33, I38, and I40.0
- Respiratory diseases: J00-J99 without J00-J06, J36, J39.0, J10-J22, J85.1, and J86
- Gastrointestinal or liver diseases: K00-K99 without K35, K37, K57.0, K57.2, K57.4, K57.8, K61, K63.0, K65.0, K65.9, K67, K75.0, K75.1, K80.0, K80.3, K80.4, K81.0, K81.9, and K83.0
- Trauma or poisoning: S00-T98
- Other: all codes not included in other categories

ICD-10 disease categories included in non-renal Charlson Comorbidity Index score
Charlson score of 1:

- Myocardial infarction: I21, I22, I23;
- Congestive heart failure: I50, I11.0, I13.0, I13.2;
- Peripheral vascular disease: I70, I71, I72, I73, I74, I77;
- Cerebrovascular disease: I60-I69, G45, G46;
- Dementia: F00-F03, F05.1, G30;
- Chronic pulmonary disease: J40-J47, J60-J67, J68.4, J70.1, J70.3, J84.1, J92.0, J96.1, J98.2, J98.3;
- Connective tissue disease: M05, M06, M08, M09, M30, M31, M32, M33, M34, M35, M36, D86;
- Ulcer disease: K22.1, K25-K28;

- Mild liver disease: B18, K70.0-K70.3, K70.9, K71, K73, K74, K76.0;
- Diabetes mellitus: E10.0-E10.2, E10.9, E11.0-E11.1, E11.9

Charlson score of 2:

- Hemiplegia: G81, G82;
- Diabetes with end organ damage: E10.2-E10.8, E11.2-E11.8;
- Any tumor: C00-C75;
- Leukemia: C91-C95;
- Lymphoma: C81-C85, C88, C90, C96

Charlson score of 3:

- Moderate to severe liver disease: B15.0, B16.0, B16.2, B19.0, K70.4, K72, K76.6, I85

Charlson score of 6:

- Metastatic solid tumor: C76-C80;
- AIDS: B21-B24

Danish treatment codes for intensive care unit treatments

- Acute dialysis: JFD0
- Mechanical ventilation: GDA0
- Treatment with inotropics or vasopressors:FHC92, FHC93, FHC95

Nordic Medico-Statistical Committee Classification of Surgical Procedures (NCSP) codes for cardiac surgery

- Heart and major thoracic vessels: KF

ICD-10 codes and Danish treatment codes for chronic renal replacement therapy and kidney transplantation

- Chronic renal replacement therapy: JFD2
- Kidney transplant status: KKAS, Z94.0
