# Supplementary material for: One-year mortality among Danish intensive care patients with acute kidney injury: a cohort study
Source: Crit Care. 2012 Jul 12;16(4):R124. doi: 10.1186/cc11420 (PMC3580703; doi:10.1186/cc11420)
Supplement: Additional file 2 — Table describing the characteristics of patients with and without a creatinine measurement on the day of ICU admission, and on the day before and the day after admission. [file cc11420-S2.PDF]

**Characteristics of patients with and without a creatinine measurement at ICU admission**

| | **Missing creatinine n = 1,578** | **Available creatinine n = 30,762** |
|---|---|---|
| **Age** | | |
| Median age (IQR) | 44 (30 ; 63) | 65 (50 ; 75) |
| **Gender** | | |
| Female | 886 (56.1%) | 13.352 (43.4%) |
| Male | 692 (43.9%) | 17,410 (56.6%) |
| **Charlson Comorbidity Index score**[a] | | |
| Low (score: 0) | 1,153 (73.1%) | 15,726 (51.1%) |
| Medium (score: 1-2) | 264 (16.7%) | 10,544 (34.3%) |
| High (score ≥3) | 161 (10.2%) | 4,492 (14.6%) |
| **Chronic kidney disease**[b] | | |
| Yes | 100 (6.3%) | 4.347 (14.1%) |
| No | 1,478 (93.7) | 26,415 (85.9%) |
| **Primary diagnose during current hospitalization** | | |
| Septicemia | 8 (0.5%) | 646 (2.1%) |
| Other infectious diseases | 162 (10.3%) | 2.959 (9.6%) |
| Endocrine diseases | 46 (2.9%) | 558 (1.8%) |
| Cardiovascular diseases | 141 (8.9%) | 8,220 (26.7%) |
| Respiratory diseases | 57 (3.6%) | 1,750 (5.7%) |
| Gastrointestinal or liver diseases | 79 (5.0%) | 3,243 (10.5%) |
| Cancer or other neoplasm | 126 (8.0%) | 3,854(12.5%) |
| Trauma or poisoning | 274 (17.4%) | 5,035 (16.4%) |
| Other | 685 (43.4%) | 4.497 (14.6%) |
| **Surgical status**[c,d] | | |
| Non-surgical | 497 (31.5%) | 11,766 (38.2%) |
| Surgical | | |
| Acute non-cardiac | 693 (43.9%) | 10,141 (33.0%) |
| Acute cardiac | 18 (1.1%) | 1,096 (3.6%) |
| Elective non-cardiac | 349 (22.1%) | 4,334 (14.1%) |
| Elective cardiac | 21 (1.3%) | 3.425 (11.1%) |
| **Laboratory information** | | |
| Measured baseline creatinine n (%) | 744 (47.2) | 21,028 (68.4%) |
| **ICU treatments** | | |
| Acute renal replacement rherapy | 16 (1.0%) | 1,469 (4.8%) |
| Mechanical ventilation | 180 (11.4%) | 12,054 (39.2%) |
| Inotropes/vasopressors | 106 (6.7%) | 10,382 (33.7%) |
| **Length of admission** | | |
| In-hospital days – median (IQR) | 3 (1 ; 7) | 10 (4 ; 21) |
| In-hospital days before ICU admission – median (IQR) | 0 (0 ; 2) | 1 (0 ; 3) |

[a] Non-renal Charlson Comorbidity Index score

[b] eGFR < 60 ml/min per $1.73m^2$

[c] Surgical status and cardiac surgical stutus identified by surgery and type of surgery on or up to 7 days before ICU admission

[d] Acute and elective status classified according to hospital admission type

AKI, acute kidney injury; CI, confidence interval; ICU, intensive care unit; IQR, inter quartile range; NA, not available
